# Double-duty actions addressing the double burden of malnutrition among adolescents aged 10–19: protocol for a scoping review

**DOI:** 10.3389/fpubh.2024.1423860

**Published:** 2024-08-12

**Authors:** Shuangyu Zhao, Sachin Shinde, Melinda Mastan, Wafaie Fawzi, Kun Tang

**Affiliations:** ^1^Vanke School of Public Health, Tsinghua University, Beijing, China; ^2^Department of Global Health and Population, T. H. Chan School of Public Health, Harvard University, Boston, MA, United States

**Keywords:** double burden of malnutrition, double-duty actions, adolescents, scoping review, protocol

## Abstract

**Background:**

The global adolescent population faces the challenge of the double burden of malnutrition (DBM), characterized by the coexistence of undernutrition and overweight/obesity, including diet-related non-communicable diseases (NCDs). This dual challenge, prevalent across various socio-economic backgrounds, necessitates double-duty actions, i.e., integrated interventions designed to concurrently address both sets of conditions. These actions are critical for fostering the overall health and well-being of adolescents. The objective of this review is to describe the content, setting, and delivery mechanisms of double-duty actions, synthesize their impacts on adolescents’ nutritional status, and offer policy and program implications for future interventions.

**Methods:**

As part of this scoping review, we will conduct a comprehensive search across multiple databases, including MEDLINE, Embase, CENTRAL, CINAHL, and Google Scholar, to identify relevant interventions, programs, policies, guidelines, evaluation studies, and strategies targeting DBM among adolescents aged 10–19 years. Inclusion criteria encompass a range of evidence sources with methodologically sound and well-described study designs. All full-text articles and abstracts will be independently screened by two reviewers to ensure a comprehensive inclusion of papers that align with the established criteria. The final protocol is available on Open Science Forum (https://osf.io/kxapb).

**Discussion:**

Addressing DBM through integrated double-duty actions is crucial for achieving global nutrition and public health goals. However, challenges persist in the form of uncoordinated efforts, lack of comprehensive evidence for what interventions work among adolescents, and the need for context-specific strategies to effectively address the heterogeneity of DBM. The results of this scoping review may provide evidence for future policies and interventions, emphasizing integrated, multi-sectoral strategies tailored to the unique needs of the adolescent population.

## Introduction

Globally, there are around 1.3 billion adolescents, comprising approximately 16% of the global population (1). Adolescence is a period with rapid physical, cognitive, and social development, which requires increased nutritional needs (2). However, this crucial developmental window also presents significant health challenges for adolescents. Currently, 8.4% of female and 12.4% of male adolescents all over the world suffer from underweight, and the prevalence has not changed much in the past 30 years (3). Concurrently, there is a sustained high prevalence of stunting, anemia, and other micronutrient deficiencies among adolescents in low- and middle-income countries (LMICs). Additionally, the global epidemic of overweight and obesity affects approximately one in every three adolescents (3). This coexistence of undernutrition and overweight, obesity, and diet-related non-communicable diseases (NCDs) has led to the term double burden of malnutrition (DBM), which can manifest at the individual, household, or population levels (4). At the individual level, DBM refers to a person who suffers from both undernutrition and overweight/obesity. At the household level, the DBM is defined as different family members suffering from multiple forms of malnutrition. DBM can also be seen at the population level if there is a high prevalence of both undernutrition and overweight/obesity within a community, region, or country (5). DBM poses new challenges for public health systems and requires integrated nutrition interventions that can address contrasting forms of malnutrition.

The prevalence of DBM remains high in countries at all levels of development, and has shown an increasing trend in recent decades driven by increases in obesity (6). A pooled analysis of 3,663 population-representative studies of 222 million individuals showed that approximately 77 million girls and 108 million boys were impacted by thinness, while 65.1 million girls and 94.2 million boys were affected by obesity in 2022. From 1990 to 2022, there was a notable increase in age-standardized prevalence of obesity, affecting 93% (186 countries) of girls and 98% (195 countries) of boys in 2022 (6). There was also significant variation in the prevalence of DBM across countries and regions, with the most substantial increase occurring in LMICs, notably in regions such as Polynesia and Micronesia, the Caribbean, and the Middle East and North Africa (6). Another study conducted on adolescents aged 12–15 years in 57 LMICs revealed that the prevalence of concurrent stunting and overweight or obesity ranged from 0.0% in Niue to 10.1% in Sri Lanka (7). These disparities underscore the critical need for nutrition interventions tailored to the specific contexts of countries and regions.

The DBM has profound health consequences for adolescent health and well-being, including increased risk of chronic diseases in adulthood, systemic inflammation, mental health problems, and poor school performance (8). The etiology of DBM is multifactorial, including biological factors, dietary patterns, socio-economic status, food environments, food systems, and governance (9). Integral to this complex interplay is the nutrition transition, driven by economic development, urbanization, and the globalization of food markets (10). The change in dietary patterns, for example, signifies a move away from nutrient-rich traditional diets toward processed foods that are calorie-dense yet nutrient-deficient. Furthermore, decreased physical activity due to more sedentary lifestyles and the mechanization of traditionally labor-intensive tasks, along with cultural perceptions and social norms about body image and food choices, also contribute to exacerbating the coexistence of undernutrition and overnutrition among adolescents (10). Addressing the multifaceted causes and consequences of DBM requires a holistic, interdisciplinary approach that encompasses not only healthcare interventions but also broader societal and policy-level changes.

Addressing DBM offers an untapped window of integrated strategies, defined as double-duty actions. Double-duty actions encompass an array of interventions, programs and policies that aim to simultaneously reduce the risk or burden of both undernutrition (including wasting, stunting and micronutrient deficiency or insufficiency) and overweight, obesity or diet-related NCDs (9). The World Health Organization (WHO) has proposed six potential shared platforms to deliver double-duty actions, including national dietary guidelines, policies, health systems, emergency nutrition programs, urban food policies and systems, and social policies (11). Nevertheless, current actions to address the different forms of malnutrition operate in silos through uncoordinated agents and platforms, which tackle single forms of malnutrition (12). Moreover, the evidence from previous reviews and meta-analyses mainly focused on under-five children, pregnant women, and newborns, neglecting the unique challenges of the DBM in adolescents population (13). A recent scoping review has summarized the double-duty actions across all age groups; however, it pursued papers in a single database and utilized a constrained searching strategy (13). To our best of knowledge, there has been no systematic synthesis of the evidence related to double-duty actions targeted at adolescents aged 10–19 years.

Double-duty actions among adolescents will be key to achieving the ambitions of the United Nations Decade of Action on Nutrition to end hunger, achieve food security and improved nutrition, and promote sustainable agriculture, and the Sustainable Development Goals to eliminate all forms of malnutrition (14). Therefore, the aim of this scoping review is to comprehensively examine the interventions, programs, policies, guidelines, strategies, and evaluation studies targeting the DBM among adolescents aged 10–19 years. More specifically, the review will describe the content of these double-duty actions that have already been designed or implemented, understand the setting of delivery mechanisms, platforms, and delivery agents of these double-duty actions, synthetize their impact on adolescents’ nutritional status, and offer policy implications for the design of future double-duty actions.

## Methods and analysis

### Data sources, search terms, and search strategy

A comprehensive search of MEDLINE (through PubMed), Embase, CENTRAL (through the Cochrane Library), CINAHL, and Google Scholar will be conducted to identify potentially eligible studies with no time limit. We aim to identify relevant studies by using the combination of medical subject headings (MeSH), keywords related to candidate double-duty actions, and shared platforms to deliver double-duty actions proposed by WHO (11). To further include additional information, we will also examine references and bibliographies of all selected studies. Our search will extend to ClinicalTrials.gov and organizational websites such as the WHO, World Bank, United Nations Children’s Fund (UNICEF), World Food Plan, and United Nations Population Fund (UNFPA). When possible, studies published in non-English languages will be translated by native speakers in the team. We will not consider studies that cannot be adequately translated.

Our search strategy is guided by the PICO format (population, interventions, comparisons, and outcomes) (Table 1). The search will be conducted following three steps. First, a broad search strategy type of study [e.g., intervention, policy, guideline, program, evaluation study] AND outcome [e.g., double burden of malnutrition] AND population [adolescents] will be conducted in PubMed, later customized to fit other databases. The effectiveness of the search strategy will be evaluated by the number of sentinel articles identified. Details of the search terms used in all databases are shown in Table 2. Secondly, for each search conducted, we will record the name of the database, date of search performed, search strategy (i.e., subject headings and keywords), filters applied, and the number of studies found. Finally, the source of studies from grey literature (i.e., conference, website, news, policy brief, etc.) or manual search will be documented.

**Table 1:** Eligibility criteria for the scoping review in PICO format.

Item	Inclusion criteria	Exclusion criteria
Population	Adolescents (10–19 years old)	Studies did not involve adolescents aged 10–19
Interventions or approaches	The interventions, programs, policies, strategies, evaluation studies, guidelines, health promotion with methodologically-sound, well-described study designs, or policy tools	Observational studies including cohort, case–control, and cross-sectional designs, or studies without methodologically-sound and well-described study designs.
Comparison	Studies that compared the intervention with any relevant control group including participants who did not receive any intervention, or those who received standard care, education, interventions	Not applicable
Outcomes	Studies should address DBM at individual- or population-level, including combined any form of undernutrition (wasting, stunting and micronutrient deficiency or insufficiency) and overweight, obesity, or diet-related NCDs	Studies involving undernutrition and overnutrition but address each form of malnutrition separately or targeted at household-level DBM

**Table 2:** Search strategy of scoping review.

No.	Concept	PubMed search terms
#1	Study design (policy, health education, health promotion, program, evaluation study, strategy, and etc)	(“Policy”[Mesh] OR “Preventive Health Services”[Mesh:NoExp] OR “Health Education”[Mesh:NoExp] OR “Health Promotion”[Mesh] OR “prevention and control” [Subheading] OR “Program Development”[Mesh] OR “Program Evaluation”[Mesh] OR “Evaluation Study” [Publication Type] OR “Food Assistance”[Mesh] OR “School Health Services”[Mesh] OR double duty [tiab] OR triple duty [tiab] OR community based[tiab] OR education[tiab] OR evaluat*[tiab] OR food aid*[tiab] OR food assistance[tiab] OR food ration*[tiab] OR food stamp*[tiab] OR guide*[tiab] OR health service*[tiab] OR health promotion*[tiab] OR health system*[tiab] OR implementation[tiab] OR initiative*[tiab] OR intervention*[tiab] OR program*[tiab] OR health promotion[tiab] OR policies[tiab] OR policy[tiab] OR prevention[tiab] OR preventive[tiab] OR recommendation*[tiab] OR school based[tiab] OR school health[tiab] OR school breakfast*[tiab] OR school feeding[tiab] OR school lunch*[tiab] OR school meal*[tiab] OR school snack*[tiab] OR strategies[tiab] OR strategy[tiab] OR regulation*[tiab] OR cash transfer* [tiab] OR school garden* [tiab] OR food system* [tiab] OR food environ* [tiab] OR approach*[tiab] OR marketing*[tiab] OR food label*[tiab])
#2	Outcome (double burden of malnutrition)	(“double burden”[tiab:~3] OR “double burdens”[tiab:~3] OR “dual burden”[tiab:~3] OR “dual burdens”[tiab:~3] OR “double trouble”[tiab:~3] OR “double troubles”[tiab:~3]) AND (“Malnutrition”[Mesh] OR “Thinness”[Mesh:NoExp] OR “Growth Disorders”[Mesh:NoExp] OR “Anemia”[Mesh] OR anemia*[tiab] OR anaemia*[tiab] OR growth disorder*[tiab] OR iron deficien*[tiab] OR malnutrition[tiab] OR malnourished[tiab] OR malnourishment[tiab] OR nutritional deficien*[tiab] OR micronutrient deficien*[tiab] OR stunted [tiab] OR stunting[tiab] OR thinness[tiab] OR undernutrition[tiab] OR undernourished[tiab] OR undernourishment[tiab] OR underweight[tiab] OR wasted [tiab] OR wasting[tiab])
#3	Population (adolescents)	(“Adolescent”[MeSH] OR adolescen*[tiab] OR teen*[tiab] OR high school*[tiab] OR highschool*[tiab] OR middle school*[tiab] OR junior high*[tiab] OR preadolescen*[tiab] OR prepupert*[tiab] OR pubert*[tiab] OR secondary school*[tiab] OR prepubescen*[tiab] OR pubescen*[tiab] OR youth[tiab] OR youths[tiab] OR young people[tiab] OR 10 years old[tiab] OR 11 years old[tiab] OR 12 years old[tiab] OR 13 years old[tiab] OR 10 years of age[tiab] OR 11 years of age[tiab] OR 12 years of age[tiab] OR 13 years of age[tiab] OR 3rd grade*[tiab] OR 4th grade*[tiab] OR 5th grade*[tiab] OR 6th grade*[tiab] OR age 10[tiab] OR age 11[tiab] OR age 12[tiab] OR age 13[tiab] OR aged 10[tiab] OR aged 11[tiab] OR aged 12[tiab] OR aged 13[tiab] OR ages 10[tiab] OR ages 11[tiab] OR ages 12[tiab] OR ages 13[tiab] OR age ten[tiab] OR age eleven[tiab] OR age twelve[tiab] OR age thirteen[tiab] OR grade 3[tiab] OR grade 4[tiab] OR grade 5[tiab] OR grade 6[tiab] OR grades 3[tiab] OR grades 4[tiab] OR grades 5[tiab] OR grades 6[tiab] OR third grade*[tiab] OR fourth grade*[tiab] OR fifth grade*[tiab] OR sixth grade*[tiab])

No.	Concept	Embase search terms
#1	Study design (policy, health education, health promotion, program, evaluation study, strategy, and etc)	(‘policy’/exp. OR ‘preventive health service’/de OR ‘health education’/de OR ‘health promotion’/exp. OR ‘prevention’) AND ‘control’ OR ‘program development’/exp. OR ‘program evaluation’/exp. OR ‘evaluation study’:it OR ‘food assistance’/exp. OR ‘school health service’/exp. OR ‘double duty’:ti,ab,kw OR ‘triple duty’:ti,ab,kw OR ‘community based’:ti,ab,kw OR ‘education’:ti,ab,kw OR ‘evaluat*’:ti,ab,kw OR ‘food aid*’:ti,ab,kw OR ‘food assistance’:ti,ab,kw OR ‘food ration*’:ti,ab,kw OR ‘food stamp*’:ti,ab,kw OR ‘guide*’:ti,ab,kw OR ‘health service*’:ti,ab,kw OR ‘health promotion*’:ti,ab,kw OR ‘health system*’:ti,ab,kw OR ‘implementation’:ti,ab,kw OR ‘initiative*’:ti,ab,kw OR ‘intervention*’:ti,ab,kw OR ‘program*’:ti,ab,kw OR ‘health promotion’:ti,ab,kw OR ‘policies’:ti,ab,kw OR ‘policy’:ti,ab,kw OR ‘prevention’:ti,ab,kw OR ‘preventive’:ti,ab,kw OR ‘recommendation*’:ti,ab,kw OR ‘school based’:ti,ab,kw OR ‘school health’:ti,ab,kw OR ‘school breakfast*’:ti,ab,kw OR ‘school feeding’:ti,ab,kw OR ‘school lunch*’:ti,ab,kw OR ‘school meal*’:ti,ab,kw OR ‘school snack*’:ti,ab,kw OR ‘strategies’:ti,ab,kw OR ‘strategy’:ti,ab,kw OR ‘regulation*’:ti,ab,kw OR ‘cash transfer*’:ti,ab,kw OR ‘school garden*’:ti,ab,kw OR ‘food system*’:ti,ab,kw OR ‘food environ*’:ti,ab,kw OR ‘approach*’:ti,ab,kw OR ‘marketing*’:ti,ab,kw OR ‘food label*’:ti,ab,kw
#2	Outcome (double burden of malnutrition)	(‘double burden’:ti,ab,kw OR ‘double burdens’:ti,ab,kw OR ‘dual burden’:ti,ab,kw OR ‘dual burdens’:ti,ab,kw OR ‘double trouble’:ti,ab,kw OR ‘double troubles’:ti,ab,kw) AND (‘malnutrition’/exp. OR ‘underweight’/de OR ‘growth disorder’/de OR ‘anemia’/exp. OR ‘anemia*’:ti,ab,kw OR ‘anaemia*’:ti,ab,kw OR ‘growth disorder*’:ti,ab,kw OR ‘iron deficien*’:ti,ab,kw OR ‘malnutrition’:ti,ab,kw OR ‘malnourished’:ti,ab,kw OR ‘malnourishment’:ti,ab,kw OR ‘nutritional deficien*’:ti,ab,kw OR ‘micronutrient deficien*’:ti,ab,kw OR ‘stunted’:ti,ab,kw OR ‘stunting’:ti,ab,kw OR ‘thinness’:ti,ab,kw OR ‘undernutrition’:ti,ab,kw OR ‘undernourished’:ti,ab,kw OR ‘undernourishment’:ti,ab,kw OR ‘underweight’:ti,ab,kw OR ‘wasted’:ti,ab,kw OR ‘wasting’:ti,ab,kw)
#3	Population (adolescents)	(‘adolescent’/exp. OR ‘adolescen*’:ti,ab,kw OR ‘teen*’:ti,ab,kw OR ‘high school*’:ti,ab,kw OR ‘highschool*’:ti,ab,kw OR ‘middle school*’:ti,ab,kw OR ‘junior high*’:ti,ab,kw OR ‘preadolescen*’:ti,ab,kw OR ‘prepupert*’:ti,ab,kw OR ‘pubert*’:ti,ab,kw OR ‘secondary school*’:ti,ab,kw OR ‘prepubescen*’:ti,ab,kw OR ‘pubescen*’:ti,ab,kw OR ‘youth’:ti,ab,kw OR ‘youths’:ti,ab,kw OR ‘young people’:ti,ab,kw OR ‘10 years old’:ti,ab,kw OR ‘11 years old’:ti,ab,kw OR ‘12 years old’:ti,ab,kw OR ‘13 years old’:ti,ab,kw OR ‘10 years of age’:ti,ab,kw OR ‘11 years of age’:ti,ab,kw OR ‘12 years of age’:ti,ab,kw OR ‘13 years of age’:ti,ab,kw OR ‘3rd grade*’:ti,ab,kw OR ‘4th grade*’:ti,ab,kw OR ‘5th grade*’:ti,ab,kw OR ‘6th grade*’:ti,ab,kw OR ‘age 10’:ti,ab,kw OR ‘age 11’:ti,ab,kw OR ‘age 12’:ti,ab,kw OR ‘age 13’:ti,ab,kw OR ‘aged 10’:ti,ab,kw OR ‘aged 11’:ti,ab,kw OR ‘aged 12’:ti,ab,kw OR ‘aged 13’:ti,ab,kw OR ‘ages 10’:ti,ab,kw OR ‘ages 11’:ti,ab,kw OR ‘ages 12’:ti,ab,kw OR ‘ages 13’:ti,ab,kw OR ‘age ten’:ti,ab,kw OR ‘age eleven’:ti,ab,kw OR ‘age twelve’:ti,ab,kw OR ‘age thirteen’:ti,ab,kw OR ‘grade 3’:ti,ab,kw OR ‘grade 4’:ti,ab,kw OR ‘grade 5’:ti,ab,kw OR ‘grade 6’:ti,ab,kw OR ‘grades 3’:ti,ab,kw OR ‘grades 4’:ti,ab,kw OR ‘grades 5’:ti,ab,kw OR ‘grades 6’:ti,ab,kw OR ‘third grade*’:ti,ab,kw OR ‘fourth grade*’:ti,ab,kw OR ‘fifth grade*’:ti,ab,kw OR ‘sixth grade*’:ti,ab,kw)

No.	Concept	CINAHL search terms
#1	Study design (policy, health education, health promotion, program, evaluation study, strategy, and etc)	(MH “Policy” OR MH “Preventive Health Services” OR MH “Health Education” OR MH “Health Promotion” OR MH “Program Development” OR MH “Program Evaluation” OR PT “Evaluation Studies” OR MH “Food Assistance” OR MH “School Health Services” OR (TI “double duty” OR AB “double duty”) OR (TI “triple duty” OR AB “triple duty”) OR (TI “community based” OR AB “community based”) OR (TI education OR AB education) OR (TI evaluat* OR AB evaluat*) OR (TI “food aid*” OR AB “food aid*”) OR (TI “food assistance” OR AB “food assistance”) OR (TI “food ration*” OR AB “food ration*”) OR (TI “food stamp*” OR AB “food stamp*”) OR (TI guide* OR AB guide*) OR (TI “health service*” OR AB “health service*”) OR (TI “health promotion*” OR AB “health promotion*”) OR (TI “health system*” OR AB “health system*”) OR (TI implementation OR AB implementation) OR (TI initiative* OR AB initiative*) OR (TI intervention* OR AB intervention*) OR (TI program* OR AB program*) OR (TI “health promotion” OR AB “health promotion”) OR (TI policies OR AB policies) OR (TI policy OR AB policy) OR (TI prevention OR AB prevention) OR (TI preventive OR AB preventive) OR (TI recommendation* OR AB recommendation*) OR (TI “school based” OR AB “school based”) OR (TI “school health” OR AB “school health”) OR (TI “school breakfast*” OR AB “school breakfast*”) OR (TI “school feeding” OR AB “school feeding”) OR (TI “school lunch*” OR AB “school lunch*”) OR (TI “school meal*” OR AB “school meal*”) OR (TI “school snack*” OR AB “school snack*”) OR (TI strategies OR AB strategies) OR (TI strategy OR AB strategy) OR (TI regulation* OR AB regulation*) OR (TI “cash transfer*” OR AB “cash transfer*”) OR (TI “school garden*” OR AB “school garden*”) OR (TI “food system*” OR AB “food system*”) OR (TI “food environ*” OR AB “food environ*”) OR (TI approach* OR AB approach*) OR (TI marketing* OR AB marketing*) OR (TI “food label*” OR AB “food label*”))
#2	Outcome (double burden of malnutrition)	((TI “double burden” OR AB “double burden” OR TI “double burdens” OR AB “double burdens” OR TI “dual burden” OR AB “dual burden” OR TI “dual burdens” OR AB “dual burdens” OR TI “double trouble” OR AB “double trouble” OR TI “double troubles” OR AB “double troubles”) N3) AND (MH “Malnutrition” OR MH “Thinness” OR MH “Growth Disorders” OR MH “Anemia” OR (TI anemia* OR AB anemia*) OR (TI anaemia* OR AB anaemia*) OR (TI “growth disorder*” OR AB “growth disorder*”) OR (TI “iron deficiency*” OR AB “iron deficiency*”) OR (TI malnutrition OR AB malnutrition) OR (TI malnourished OR AB malnourished) OR (TI malnourishment OR AB malnourishment) OR (TI “nutritional deficiency*” OR AB “nutritional deficiency*”) OR (TI “micronutrient deficiency*” OR AB “micronutrient deficiency*”) OR (TI stunted OR AB stunted) OR (TI stunting OR AB stunting) OR (TI thinness OR AB thinness) OR (TI undernutrition OR AB undernutrition) OR (TI undernourished OR AB undernourished) OR (TI undernourishment OR AB undernourishment) OR (TI underweight OR AB underweight) OR (TI wasted OR AB wasted) OR (TI wasting OR AB wasting))
#3	Population (adolescents)	(MH “Adolescents” OR (TI adolescen* OR AB adolescen*) OR (TI teen* OR AB teen*) OR (TI “high school*” OR AB “high school*”) OR (TI highschool* OR AB highschool*) OR (TI “middle school*” OR AB “middle school*”) OR (TI “junior high*” OR AB “junior high*”) OR (TI preadolescen* OR AB preadolescen*) OR (TI prepupert* OR AB prepupert*) OR (TI pubert* OR AB pubert*) OR (TI “secondary school*” OR AB “secondary school*”) OR (TI prepubescen* OR AB prepubescen*) OR (TI pubescen* OR AB pubescen*) OR (TI youth OR AB youth) OR (TI youths OR AB youths) OR (TI “young people” OR AB “young people”) OR (TI “10 years old” OR AB “10 years old”) OR (TI “11 years old” OR AB “11 years old”) OR (TI “12 years old” OR AB “12 years old”) OR (TI “13 years old” OR AB “13 years old”) OR (TI “10 years of age” OR AB “10 years of age”) OR (TI “11 years of age” OR AB “11 years of age”) OR (TI “12 years of age” OR AB “12 years of age”) OR (TI “13 years of age” OR AB “13 years of age”) OR (TI “3rd grade*” OR AB “3rd grade*”) OR (TI “4th grade*” OR AB “4th grade*”) OR (TI “5th grade*” OR AB “5th grade*”) OR (TI “6th grade*” OR AB “6th grade*”) OR (TI “age 10” OR AB “age 10”) OR (TI “age 11” OR AB “age 11”) OR (TI “age 12” OR AB “age 12”) OR (TI “age 13” OR AB “age 13”) OR (TI “aged 10” OR AB “aged 10”) OR (TI “aged 11” OR AB “aged 11”) OR (TI “aged 12” OR AB “aged 12”) OR (TI “aged 13” OR AB “aged 13”) OR (TI “ages 10” OR AB “ages 10”) OR (TI “ages 11” OR AB “ages 11”) OR (TI “ages 12” OR AB “ages 12”) OR (TI “ages 13” OR AB “ages 13”) OR (TI “age ten” OR AB “age ten”) OR (TI “age eleven” OR AB “age eleven”) OR (TI “age twelve” OR AB “age twelve”) OR (TI “age thirteen” OR AB “age thirteen”) OR (TI “grade 3” OR AB “grade 3”) OR (TI “grade 4” OR AB “grade 4”) OR (TI “grade 5” OR AB “grade 5”) OR (TI “grade 6” OR AB “grade 6”) OR (TI “grades 3” OR AB “grades 3”) OR (TI “grades 4” OR AB “grades 4”) OR (TI “grades 5” OR AB “grades 5”) OR (TI “grades 6” OR AB “grades 6”) OR (TI “third grade*” OR AB “third grade*”) OR (TI “fourth grade*” OR AB “fourth grade*”) OR (TI “fifth grade*” OR AB “fifth grade*”) OR (TI “sixth grade*” OR AB “sixth grade*”))

No.	Concept	Cochrane search term
#1	Study design (policy, health education, health promotion, program, evaluation study, strategy, and etc)	#1MeSH descriptor: [Policy] explode all trees #2MeSH descriptor: [Preventive Health Services] explode all trees #3MeSH descriptor: [Health Educators] explode all trees #4MeSH descriptor: [Health Promotion] explode all trees #5MeSH descriptor: [Program Development] explode all trees #6MeSH descriptor: [Program Evaluation] explode all trees #7(“evaluation study”):pt. #8(prevention and control):kw #9MeSH descriptor: [Food Assistance] explode all trees #10MeSH descriptor: [School Health Services] explode all trees #11(double duty):ti OR (double duty):ab OR (triple duty):ti OR (triple duty):ab #12(“community based”):ti OR (“community based”):ab OR (education):ti OR (education):ab #13(evaluat*):ti OR (evaluat*):ab #14(food aid*):ti OR (food aid*):ab OR (food assistance):ti OR (food assistance):ab #15(food ration*):ti OR (food ration*):ab OR (food stamp*):ti OR (food stamp*):ab #16(guide*):ti OR (guide*):ab OR (health service*):ti OR (health service*):ab #17(health promotion*):ti OR (health promotion*):ab OR (health system*):ti OR (health system*):ab #18(implementation):ti OR (implementation):ab OR (initiative*):ti OR (initiative*):ab #19(intervention*):ti OR (intervention*):ab OR (program*):ti OR (program*):ab #20(policies):ti OR (policies):ab OR (policy):ti OR (policy):ab #21(prevention):ti OR (prevention):ab OR (preventive):ti OR (preventive):ab #22(recommendation*):ti OR (recommendation*):ab OR (school based):ti OR (school based):ab #23(school health):ti OR (school health):ab OR (school breakfast*):ti OR (school breakfast*):ab #24(school feeding):ti OR (school feeding):ab OR (school lunch*):ti OR (school lunch*):ab #25(school meal*):ti OR (school meal*):ab OR (school snack*):ti OR (school snack*):ab #26(strategies):ti OR (strategies):ab OR (strategy):ti OR (strategy):ab #27(regulation*):ti OR (regulation*):ab OR (cash transfer*):ti OR (cash transfer*):ab #28(school garden*):ti OR (school garden*):ab OR (food system*):ti OR (food system*):ab #29(food environ*):ti OR (food environ*):ab OR (approach*):ti OR (approach*):ab #30(marketing*):ti OR (marketing*):ab OR (food label*):ti OR (food label*):ab #31{OR #1-#30}
#2	Outcome (double burden of malnutrition)	#32(double burden):ti OR (double burden):ab OR (double burdens):ti OR (double burdens):ab #33(dual burden):ti OR (dual burden):ab OR (dual burdens):ti OR (dual burdens):ab #34(double trouble):ti OR (double trouble):ab OR (double troubles):ti OR (double troubles):ab #35{OR #32-#34} #36MeSH descriptor: [Malnutrition] explode all trees #37MeSH descriptor: [Thinness] explode all trees #38MeSH descriptor: [Growth Disorders] explode all trees #39MeSH descriptor: [Anemia] explode all trees #40(anemia*):ti OR (anemia*):ab OR (anaemia*):ti OR (anaemia*):ab #41(growth disorder*):ti OR (growth disorder*):ab OR (iron deficiency*):ti OR (iron deficiency*):ab #42(malnutrition):ti OR (malnutrition):ab OR (malnourished):ti OR (malnourished):ab #43(“malnourishment”):ti OR (malnourishment):ab OR (nutritional deficiency*):ti OR (nutritional deficiency*):ab #44(micronutrient deficiency*):ti OR (micronutrient deficiency*):ab OR (stunted):ti OR (stunted):ab #45(stunting):ti OR (stunting):ab OR (thinness):ti OR (thinness):ab #46(undernutrition):ti OR (undernutrition):ab OR (undernourished):ti OR (undernourished):ab #47(undernourishment):ti OR (undernourishment):ab OR (underweight):ti OR (underweight):ab #48(wasted):ti OR (wasted):ab OR (wasting):ti OR (wasting):ab #49{OR #36-#48} #50#35 AND #49
#3	Population (adolescents)	#51MeSH descriptor: [Adolescent] explode all trees #52(adolescen*):ti OR (adolescen*):ab OR (teen):ti OR (teen):ab #53(high school*):ti OR (high school*):ab OR (highschool*):ti OR (highschool*):ab #54(middle school*):ti OR (middle school*):ab OR (junior high):ti OR (junior high):ab #55(preadolescen*):ti OR (preadolescen*):ab OR (prepupert*):ti OR (prepupert*):ab #56(pubert*):ti OR (pubert*):ab OR (secondary school):ti OR (secondary school):ab #57(prepubescen*):ti OR (prepubescen*):ab OR (pubescen*):ti OR (pubescen*):ab #58(youth):ti OR (youth):ab OR (young people):ti OR (young people):ab #59(10 years old):ti OR (10 years old):ab OR (11 years old):ti OR (11 years old):ab #60(12 years old):ti OR (12 years old):ab OR (13 years old):ti OR (13 years old):ab #61(10 years of age):ti OR (10 years of age):ab OR (11 years of age):ti OR (11 years of age):ab #62(12 years of age):ti OR (12 years of age):ab OR (13 years of age):ti OR (13 years of age):ab #63(3rd grade):ti OR (3rd grade):ab OR (4th grade):ti OR (4th grade):ab #64(5th grade):ti OR (5th grade):ab OR (6th grade):ti OR (6th grade):ab #65(age 10):ti OR (age 10):ab OR (age 11):ti OR (age 11):ab #66(age 12):ti OR (age 12):ab OR (age 13):ti OR (age 13):ab #67(aged 10):ti OR (aged 10):ab OR (aged 11):ti OR (aged 11):ab #68(aged 12):ti OR (aged 12):ab OR (aged 13):ti OR (aged 13):ab #69(ages 10):ti OR (ages 10):ab OR (ages 11):ti OR (ages 11):ab #70(ages 12):ti OR (ages 12):ab OR (ages 13):ti OR (ages 13):ab #71(age ten):ti OR (age ten):ab OR (age eleven):ti OR (age eleven):ab #72(age twelve):ti OR (age twelve):ab OR (age thirteen):ti OR (age thirteen):ab #73(grade 3):ti OR (grade 3):ab OR (grade 4):ti OR (grade 4):ab #74(grade 5):ti OR (grade 5):ab OR (grade 6):ti OR (grade 6):ab #75(grades 3):ti OR (grades 3):ab OR (grades 4):ti OR (grades 4):ab #76(grades 5):ti OR (grades 5):ab OR (grades 6):ti OR (grades 6):ab #77(third grade):ti OR (third grade):ab OR (fourth grade):ti OR (fourth grade):ab #78(fifth grade):ti OR (fifth grade):ab OR (sixth grade):ti OR (sixth grade):ab #79{OR #51-#78}

### Eligibility

The inclusion and exclusion criteria for our scoping review are listed below.

#### Inclusion criteria

We will include the following studies:

- Types of evidence sources: Interventions (randomized controlled trials (RCT) and quasi-experimental studies), programs, policies, guidelines, evaluation studies, and strategies with methodologically-sound, well-described study designs, or policy tools will be included in the scoping review.
- Types of participants: Studies should be conducted in adolescent boys and girls aged 10–19 years, as defined by the WHO (15), or the age range of the participants overlapped with this window.
- Types of interventions: Studies involving actions for one or more of the following: diet-related policy, nutrition guidelines, diet recommendations, interventions to promote healthy diets, food stamps, cash transfer, school/community gardens, food system/environment interventions, social marketing, food labeling interventions, food aid, feeding programs.
- Outcome measures: The outcome will be the burden or risk of double burden of malnutrition at individual or population levels, including combined form of undernutrition (stunting, wasting, underweight or thinness, micronutrient deficiencies) and overweight, obesity, or diet-related NCDs.
- Comparison: The control group can be participants who did not receive any intervention, or those who received standard care, or education interventions.
- We will not place any restrictions on the language, publication year, sample size, or duration of the intervention.

#### Exclusion criteria

We will not consider the following studies:

- Studies did not involve adolescents aged 10–19 years.
- Observational studies including cohort, case–control, and cross-sectional designs, or studies without methodologically sound and well-described study designs.
- Studies targeted DBM at household-level or did not address DBM.

### Data management

All materials will be imported into Covidence, a web-based platform for managing systematic reviews developed by Veritas Health Innovation, Melbourne, Australia. We will identify and remove the duplicate records, as well as the screening of titles, abstracts, and full texts on Covidence.

### Selection of studies

The selection of studies will be conducted on Covidence. Firstly, titles and abstracts of imported studies will be independently evaluated by two reviewers to exclude studies that do not align with inclusion and exclusion criteria. Next, two reviewers will independently assess the full texts of the remaining studies using the same criteria. Any disagreements will be discussed between the reviewers, and if consensus cannot be reached, a third reviewer will be consulted to make the final decision. We will generate a study flow diagram to document the reasons for exclusions, following the Preferred Reporting Items for Systematic Reviews and Meta Analyses—Extension for Scoping Reviews (PRISMA-ScR) (16).

### Data extraction

Two reviewers will independently extract needed information from the included. To ensure consistency and accuracy, we will create a data extraction form based on five randomly selected studies. The data extraction process will include:

- Study information: title, authors (first and corresponding), contact information of the corresponding author, publication source, publication year, year of intervention conducted, country, and funding agency.
- Methods: objective of the research/research question, type of study, settings (school-based or community-based), sample size, and sample characteristics (age, sex, socioeconomic status, etc.).
- Interventions: delivery platform, delivery mechanisms, delivery agents, theory/framework/guidelines utilized to design the study, types of interventions, intervention duration, intervention description, and comparator/control.
- Outcome measures: measurement tools and assessed outcomes.
- Results: facilitators and challenges to the implementation, acceptance of the intervention, the coverage of services, effectiveness (including point estimate, 95% confidence intervals, *p*-values, etc.), and any other relevant findings.

We will reach out to the corresponding author via email if there is any missing or unclear information, limiting our inquiries to two times. We will analyze the available and discuss any gaps due to missing data if the data issue cannot be resolved after reaching out to the authors.

### Synthesis of evidence

We will undertake a comprehensive synthesis of all included studies and describe the findings in text and tables, following the SWiM guidelines (Synthesis Without Meta-analysis) (17). The synthesis procedure will include the number of sources, number of papers screened, evaluated for eligibility, and finally included in scoping review, with explicit reasons for exclusion at each phase. The integrated evidence will be systematically organized, including summary characteristics, citations, and critical evaluations as needed. The studies will be categorized based on study type, intervention focus, delivery agents, delivery mechanisms, and measured outcomes. The synthesis will also explore the methods of synthesis, results reporting, reliability of the results, heterogeneity in effects, and the challenges and facilitators of intervention implementation. For continuous outcomes, we will report effect sizes as mean differences (with 95% confidence intervals if available) between the intervention and control groups. For categorical outcomes, effect size will be reported as risk ratios, incident risk ratios, hazard ratios, or odds ratios (with 95% confidence intervals if available). Furthermore, in this review we will discuss the limitations arising in review process, interpret the findings in line with the objectives of the review, and provide potential policy implications and future research directions on double-duty actions. Our method will be guided by the PRISMA-ScR checklist to ensure a replicable and robust process (16).

### Registration and reporting

The final protocol has been registered in advance on the Open Science Forum, available at https://osf.io/kxapb on April 25, 2024, based on the PRISMA-ScR. We will systematically record the date of each change, describe the modifications made, and provide the rationale behind changes on the Open Science Forum.

## Discussion

Adolescents have unique nutritional needs due to their rapid physical, cognitive and social growth and development (3). However, the double burden of malnutrition, where adolescents experience both undernutrition and overnutrition, presents a complex set of in many LMICs (4). Differentiating between undernutrition (stunting, wasting) and overweight and obesity in adolescents can be difficult due to limited data collection and the coexistence of both issues. Moreover, the causes of the double burden are complex and intertwined (8). Poverty, lack of access to diverse, nutritious foods, and unhealthy food environments high in processed foods and sugary drinks all play a role. Further, LMICs often have limited resources for implementing and sustaining comprehensive nutrition interventions. A lack of trained healthcare workers can hinder the development and implementation of effective programs.

Double-duty actions hold significant potential for adolescents by ensuring adolescents get the nutrients they need to prevent undernutrition and deficiencies like iron deficiency anemia, a common issues, while also reducing the risk of overweight and obesity, which are on the rise globally (9). However, the effectiveness of double-duty actions can vary across different cultural and demographic contexts, which requires tailored interventions that consider local customs and dietary practices. Additionally, fragmented governance and lack of intersectoral collaboration currently hinder the development and implementation of integrated double-duty actions (18). The lack of high-quality evaluation studies on the long-term effectiveness of double-duty actions also complicates advocacy for their widespread adoption and funding (19, 20). This scoping review aims to map and synthesize existing evidence on double-duty actions that address DBM in adolescents aged 10–19 years. We aim to achieve a comprehensive understanding of the existing interventions, policies, guidelines, and evaluation studies. This will include assessing the delivery mechanism employed by these programs and interventions. Furthermore, we will identify context-specific strategies double-duty actions that are effective within unique demographic and cultural contexts of different regions and countries. By highlighting the need for a shift from traditional, single-domain interventions toward integrated double-duty actions, this review will provide valuable evidence to inform public health policies and research.

### Ethics and dissemination

As our study is a scoping review that analyzes the existing literature, it does not require formal ethics approval. The focus of the review is to provide a summary of double-duty actions targeting DBM among adolescents aged 10–19 years. The findings of this review will be shared through academic publications in peer-reviewed journals and presentations at both international and national conferences, targeting at researchers, policymakers, and governmental agencies. The aim is to share knowledge on effective interventions, identify gaps and disparities among double-duty actions, and offer insights that can assist policymakers to develop, refine, and propose future double-duty actions among adolescents.
